# Lung cancer screening with AI can discover cures for many early diseases. A public utility can make sure it happens

**DOI:** 10.3389/fonc.2026.1797777

**Published:** 2026-03-10

**Authors:** James L. Mulshine, Bruce S. Pyenson

**Affiliations:** 1 Department of Internal Medicine, Rush University, Chicago, IL, United States; 2 Pyenson Healthcare Analytics, LLC, Madison, NJ, United States

**Keywords:** algorithm validation, artificial intelligence, chest CT, coronary artery calcification, emphysema, lung cancer screening, public utility

## Abstract

Many nations around the world are now implementing CT-based lung cancer screening. Growing evidence led the United States to require insurance coverage for LCS in high-risk individuals. Current CT scanners can obtain vast amounts of anatomic and quantitative information from the viscera of the chest cavity, and it has become evident that the CT images obtained from LCS contain additional health information, including information that enables the early detection of other major tobacco-associated diseases, such as coronary artery disease and emphysema. Chest CT screening is now being integrated with the use of AI tools, and such tools will be essential to organize and manage the complex screening workflow required to efficiently deliver this rapidly expanding service.

A threat to realizing the health benefits of chest CT screening is the difficulty in aggregating sufficient numbers of CT images and clinical follow-up data for research purposes. Enabling access to clinical imaging and outcome data as a public utility may be essential in addressing bottlenecks to innovation to early chest disease management. The collections of chest CT images with clinical data that are being accrued for routine screening care could be repurposed with web-based strategies at low cost to enable a new range of strategic analyses and rapid AI tool development.

## Introduction

Lung cancer remains the world’s most lethal cancer (1). Fortunately, lung cancer screening has objectively been shown to reduce lung cancer mortality outcomes in long term follow-up (2–4). Detection of early-stage lung cancer before disease symptoms generally results in curative outcomes from either minimally invasive surgery or radiotherapy with modest risk of untoward treatment side effects. Lung cancer implementation is ongoing internationally with preservation of mortality benefit (5). The pace of uptake of this screening process has not been robust and measures to increase uptake have not yet been successful (6). A concern exists that the full benefits of chest CT screening have not been communicated to either the medical community or the public. While considerable enthusiasm exists about the potential for public health benefit with the chest CT screening detection of additional, major co-morbid tobacco-related diseases, lung cancer screening may provide other significant public health benefits that have not yet been considered (7, 8).

With best-practice lung cancer screening, the chest CT imaging data as well as clinical follow-up information is preserved, which allows the detection of lung cancers that may emerge over years of sequential screening images. Recent publications have already used such image collections for further optimizing screening care and developing or validating artificial intelligence (AI) tools among other reasons (9, 10).

The potential of real-world data, such as images from LCS, to support clinical innovation is attracting attention from regulators. Former Food and Drug Administration Commissioner, Dr. Robert Califf recently called for a fundamental reconsideration of evidence sources including electronic health record information to accelerate healthcare innovation (11).

With ongoing innovations within CT technology, image quality continues to improve while medical radiation doses continue to decrease (12). This dynamic may improve LCS participation. However, even with slow uptake, global implementation of screening will result in millions of screening participants and LCS will soon generate millions of CT images. Enabling open-research access to large collections of chest CT images with associated clinical outcome data acquired from routine LCS is feasible and could accelerate and sustain the development of life-saving medical interventions (13, 14). Access to such an open research resource could empower a large community of innovators to develop and validate the reliability of relevant next generation AI tools at low cost. This resource could also be used for inexpensive and comprehensive chest CT imaging quality analysis of precision to reduce variability (15, 16). In this Perspective, the authors propose the creation of public utilities that will create and facilitate routine research access to huge LCS imaging/clinical registries.

## Current status of research with LCS images

The utility of a chest CT registry is already evident. Upon completion of the NLST, 48,547 chest CT images and associated clinical outcomes data acquired through the trial, were made available for open research (17). This data as been used to estimate the potential to detect other tobacco-related diseases including atherosclerotic heart disease and emphysema (18, 19). The usual limitations of using screening trial data for population health purposes affects these data, including long time lags for curation and the selection biases associated with screening trial recruitment. In addition, rapid progression of CT technology quickly limits the value of older collections (17). The NLST images were acquired starting 2002 and typically used 4-detector scans with up to 2.5mm slice thickness. As a result, this CT image collection is now obsolete for many studies.

To optimally manage the routine ongoing care of screening subjects, all annually acquired chest CT screening images already are being stored (along with relevant clinical outcomes data) at the institution providing the screening care. We and others have reported that a cloud-based environment with appropriate permissions can interrogate that already stored imaging and clinical data at the host imaging site. This approach allows computational analyses of images from multiple sites in a secure, economical and efficient fashion (13, 14).

### The value of a public utility for imaging and clinical data resource

The authors propose the creation of public utilities for screening images and associated clinical data that can be accessed and updated efficiently and economically using current cloud computing. Existing medical image and data cloud storage environments maintained with robust data security provisions mean that access to this screening participant data resource can be secure (13, 14). For several reasons including promoting the public good, the authors propose establishing a public utility model rather than defaulting to the traditional private equity model. With appropriate governance, the public utility model (public library) could also better mitigate public skepticism while ensuring equity in participation and accessibility (20, 21).

AI tool development and validation require relevant material which constitutes “ground truth.” Under a traditional approach, for imaging biomarkers, this would require that a large number of screening participants agree to provide access to their chest CT images and clinical information. Ideally, this process requires a regular flow over years of such data, since imaging and corresponding AI tools continue to rapidly evolve. Therefore, having a stable but dynamic source of imaging/clinical data is ideal for building AI tools and maintaining data provenance while accelerating tool development. However, the recruitment of participants under the traditional approach would be burdensome and expensive.

There is an urgent need for process efficiencies in the global implementation of screening. Currently, any chest CT scanner used for screening can acquire a vast amount of relevant imaging data, and the deployment of this new service could overwhelm today’s professional radiological workflow. Therefore, developing tools, especially using AI, are needed to assist radiologist with chest CT screening review.

A particular need with tool development is to assist radiologists in performing computational analysis of serial annual chest CT scans to follow the status of disease progression in a screening participant to ensure productive early detection of potentially lethal chest diseases.

Systematically monitoring strategic imaging biomarkers in a fraction of the images potentially available through an LCS public utility could provide a powerful public health tool to optimize clinical management and to systematically assess the health of nations.

### Emerging clinical benefits with coronary calcium detection on screening chest CT

A number of investigators have reported that chest CT imaging to measure coronary vascular calcifications is the most informative biomarker to manage the risk of cardiac mortality, often before the development of symptoms of heart disease (22, 23). Recommended interventions—such as lifestyle and statin administration are already used to mitigate risk in this setting with evidence that chest CT imaging does a better job of assessing cardiovascular risk than measuring LDL or any other lipid biomarker (22–24). CT-screen-detected coronary artery disease is already recognized by cardiology professional societies, with guideline recommendations for management with statins, which are low-cost generics. Further improvement in screening participants’ health can occur since information obtained from LCS can detect the presence of coronary calcium which might provide additional motivation to adhere to lifestyle interventions such as with smoking cessation or increased physical activity (7, 8, 24).

### Emerging clinical benefits with detection of emphysema

The ability to detect emphysema in LCS scans has already been reported based on retrospective analysis of NLST archival data (19). Asymptomatic screening subjects may benefit from more intensive smoking cessation interventions today with a range of promising pharmaceutical interventions for early lung disease moving forward (24).

### Additional chest conditions detected on screening CT

**Figure 1:**
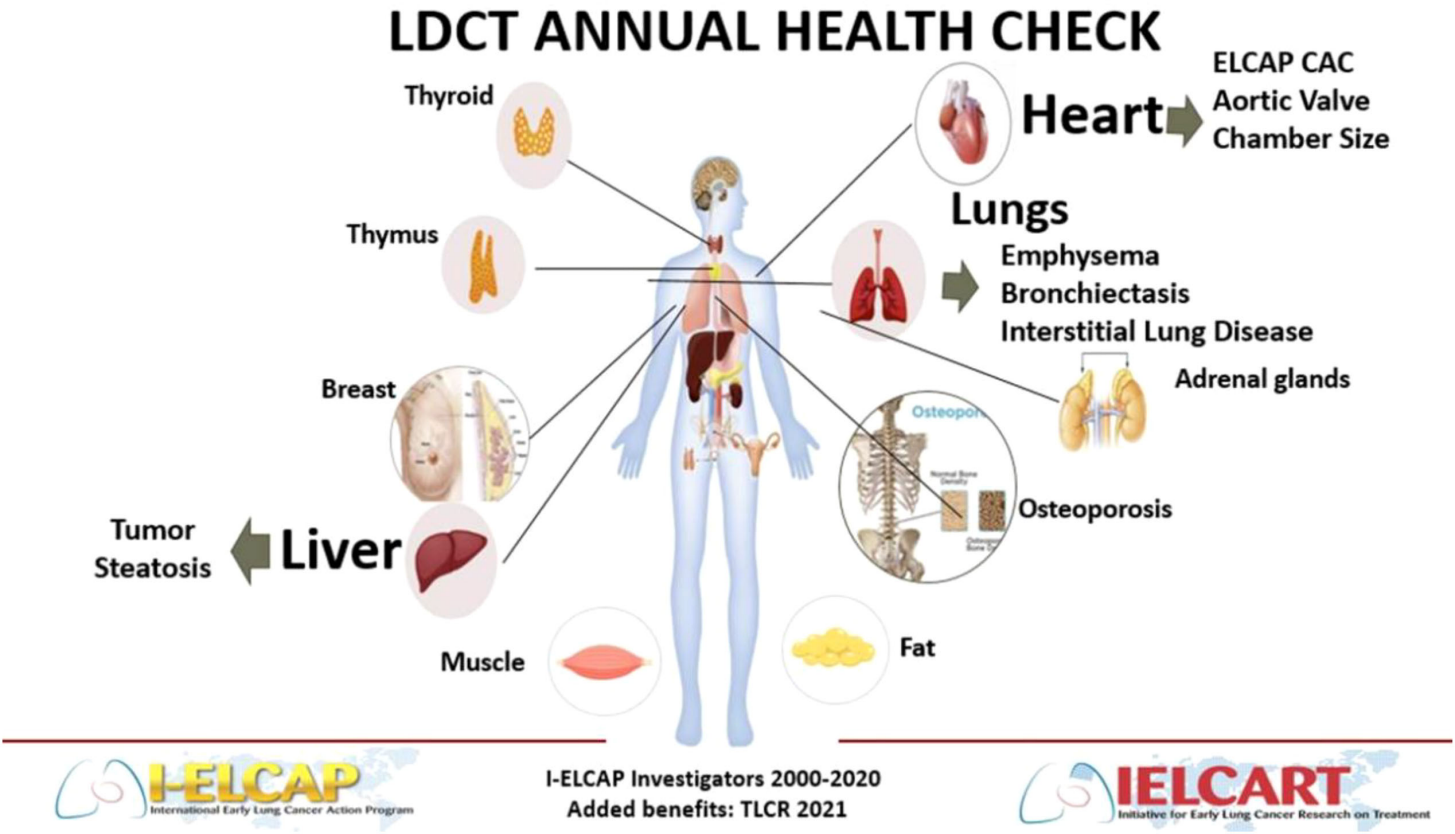
Sites where disease entities can be detected with chest CT screening.

Moving beyond the benefits we can achieve today, there are many serious conditions of the chest where we need better tools and predictions. Some of these are relatively common conditions, such as emphysema or atrial fibrillation, while others are rare, such as particular forms of interstitial lung disease. Today, CT scans often play a role in evaluating these conditions among high-risk or symptomatic patients (22–24). In **Figure 1**, a schematic developed based on the work of the International-Early Lung Cancer Action Project (I-ELCAP) highlights a number of chest diseases which can be visualized on a thoracic CT scan (24). As AI matures, we may learn to discriminate subtle patterns recognized on chest CT that will predict who is most likely to develop these or related conditions, perhaps years earlier than is currently possible. This will be possible with the proposed public utility partly because AI can identify relationships that are unexpected, but also because the public utility will allow researchers to follow large numbers of screening participants for years, which would be impractical with current, manual approaches.

### Barriers to sustained innovation with imaging

Researchers using AI or other big-data analytic approaches for tool development may need access to huge libraries of images from diverse settings and people, especially when developing tools for public health activities like screening. Currently, collecting but, today, collecting images is expensive and slow, and today, most AI tools have been trained or tested on mere hundreds of images, often from a single academic institution, and perhaps only using images from a single type of CT scanner. Another barrier to progress is the proprietary nature of most existing defined purpose biomedical image collection which may be related to high prices demanded by major institutions for their image contributions, which means that well-capitalized private firms have a huge advantage. The proprietary nature of most biomedical image collection is another barrier to progress. These collections may have narrowly-defined purposes and limited volumes, which may reflect the high prices demanded by major institutions for their image collections. These constraints mean that well-capitalized private firms have huge advantages over other researchers. After developing and launching AI tools using private image collections, private capital financed firms have little incentive to either make their image collections available for other AI development or to update their tools.

For the last several decades, the hardware side of imaging (e.g., CT scanner) has rapidly evolved, fueled by improvements in microprocessor capabilities, detector sensitivity, cloud computing and the development of AI (7, 8, 12). Since all these components interact, this constant component evolution means that the relationship between hardware and AI tools will remain dynamic, especially as the innovation cycle shortens. For example, improving imaging detail may enable even earlier detection of disease states and allow for less aggressive curative intervention which might result in an innovation that may improve a specific health outcome.

However, any change in an imaging component, such as improvements in image detectors or analysis capabilities, requires re-validation of tools to ensure the consistency of quality performance. To allow such validation cycles to be completed rapidly, routine access to appropriate image/data cases is required. Sustaining innovation in this important new screening setting drives urgency in defining approaches that provide large numbers of cases for image/data libraries. The dynamic nature of AI tool development in medical imaging will necessitate ongoing collection of new clinical, data images to sustain progress in early chest disease outcomes.

## Proposed solutions

We offer a hypothetical (but realistic) example of how the public utility/AI might develop a product. Based on analyzing tens of thousands of historical cases accumulated over several years, a team of researchers might conclude that particular places in the lung, with certain patterns of change (barely visible to a radiologist), are at high risk of developing aggressive lung cancer. This finding would be published and incorporated into the image review software that radiologists use, and the software would flag images with the suspicious pattern for radiologist scrutiny. Recommended follow-up would be guided by the outcomes observed in the historical cases. Perhaps the individual will need re-scanning at 6-month intervals instead of annually, or the individual might be referred for additional workup.

### Models of success with image and outcome data collection

Assembling the required volume of images associated with clinical outcomes to enable open-access research faces a number of obstacles. These include skepticism of screening participants about how their CT scans may be used, concerns over the high costs of curating the collected scans, and ensuring confidential access over the collection’s lifetime. Fortunately, there has been important progress in solving obstacles of privacy, data manipulation, procurement, and ownership.

Nonprofit research efforts have made excellent progress despite suffering from limited funds and limited access to medical images and related clinical data. Several organizations have laid the organizational and technical foundation for the public service utility model and examples include the Open-Source Imaging Consortium for Interstitial Lung Disease which has catalyzed progress with the spectrum of pulmonary fibrotic diseases (25). A growing inventory of valuable medical images is being assembled and curated by The Medical Imaging and Data Resource Center, which is working to create globally harmonized processes to support open research for medical imaging (26). Another image collection has been developed by the International-Early Lung Cancer Action Project (I-ELCAP), which is the largest and most influential of lung cancer screening organizations, with over 200,000 chest CT images collected longitudinally for more than 25 years (4, 10, 24). The leaders of these organizations have actively developed rigorous quality assurance processes, and the current image quality of the collections is excellent.

There are numerous examples of patient data sharing. The Regional Health Information Organizations (RHIOs, e.g., The Bronx Regional RHIO) are useful precedents (27). RHIOs attempt to fix healthcare fragmentation by gathering a patient’s medical and treatment information from multiple providers and assemble it to help a provider treat that patient. In some ways, our proposed Utility is simpler because its focus is research or process improvement, not providing real-time information to the provider treating the patient.

### Technology progress

Organizations such as the Quantitative Imaging Biomarker Alliance (QIBA) of the Radiological Society of North America have demonstrated important technical and workflow approaches to the assemble, curate, and evaluate such large numbers of digital medical images to enable process optimization on measuring imaging biomarkers within defined constraints (28). Their publications have proven the feasibility and utility of many of the recommended acquisition parameters allowing robust computer analysis especially for measuring feature such as volume consistently. A QIBA profile providing guidance with measurement issues for chest CT lung cancer screening has been published to support more reliable nodule volume quantitation (29).

Others have contributed to the understanding of how cloud-based federated image and data collection can comply with the national and international regulations, such as the European General Data Protection Regulation (11, 13, 14).

### Progress in ethical framework

A framework supporting the ethics of public ownership of images as a health imperative to enable responsible AI progress has also been developed (30). This framework supports our vision of creating a public service utility (library) to assemble, curate, and facilitate AI and other research on donated images. Larson and colleagues have also suggested an ethical framework for more nimbly sharing de-identified clinical data for AI development that frames data sharing as a public good and holds data users as data stewards with fiduciary responsibility to safeguarding the privacy of donors’ data when developing health-directed AI tools (31).

## How a public utility might work

US regulations for today’s public utilities (water, electric, etc.) date back to the 19^th^ century, but there is a significant movement to update these regulations for the technological realities of the 21st century (32). We propose that a public utility could “house” a virtual collection of LCS images and associated clinical information. The actual information would continue to be stored at the screening participant care sites, as physical access in one location is not needed for relevant research. There are many ways to implement this public utility, including, for example, a regional approach (state-based in a US context).

A US state-chartered corporation could have enabling legislation that would require that providers allow the utility to access images and related data from LCS delivered in the state. We see analogues in the state Maternal Mortality Review Committees, which have operated for almost a century to track pregnancy-related deaths (33). Some states already use provider taxes to fund public health efforts (34), and this could be a viable way to fund the public utility. Requirements for data capture, quality, and participation would be imposed on providers, and rules for research access (including fees) would be established. As states establish utilities, data sharing collaboratives could be established for multi-state efficiencies (35).

Even a smaller state can generate meaningful databases. For example, Kentucky with about 4.6 million lives has about 1.25% of the US population. If, the US generates 2 million LCS scans in a year, Kentucky, on a pro-rata basis could generate about 25,000 images per year. New York, a larger state with 20 million lives, or about 5.5% of the US population, could generate over 100,000 images. With such an approach, robust image repositories could be accreted and economically maintained as with the IASLC cloud-based proof-of-concept example (14).

**Figure 2:**
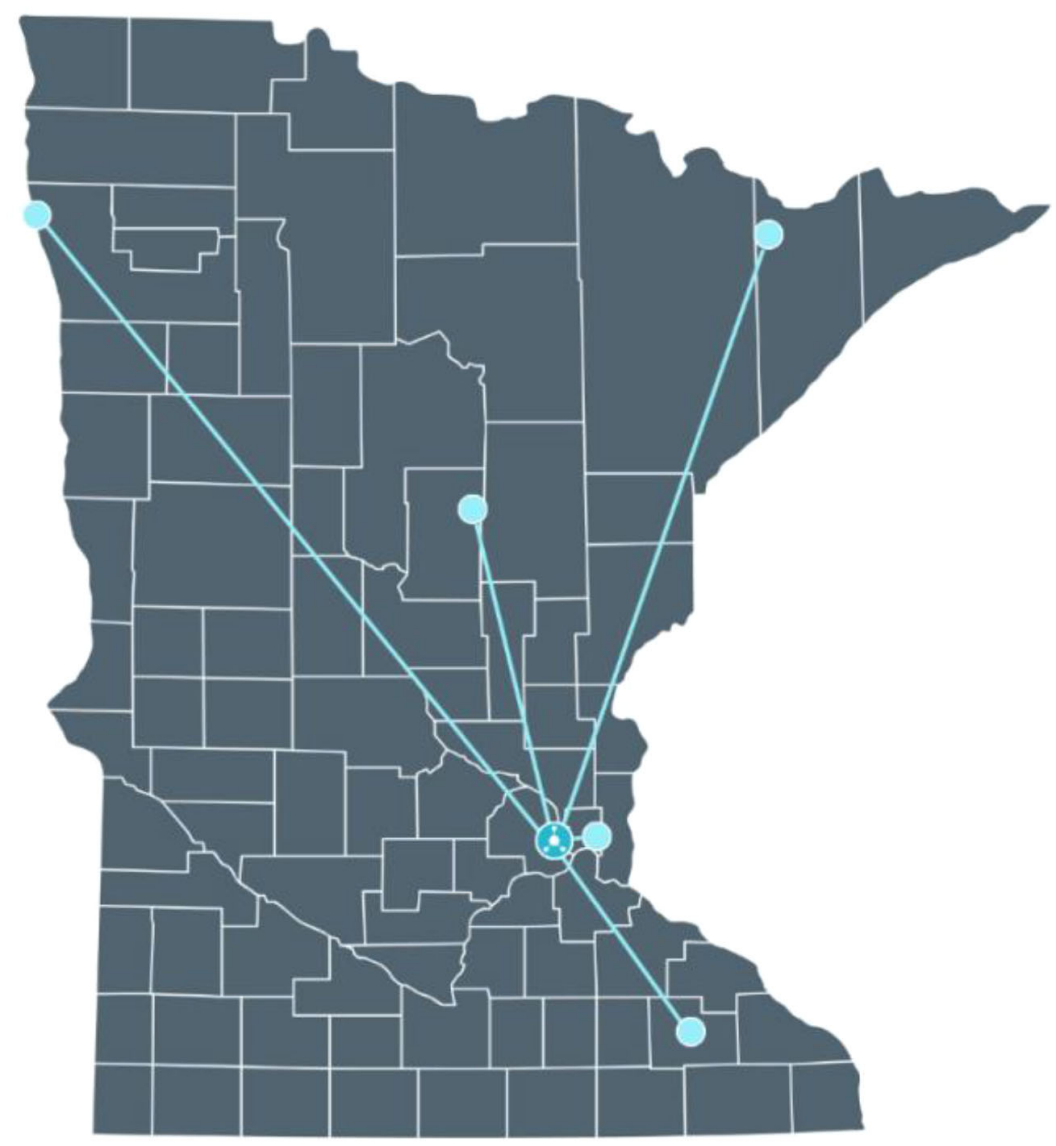
Schematic of a possible state-wide cloud-based network of screening sites which allow access to cloud delivered analysis software for chest CT images and associated, de-identified metadata. Illustration of a possible hub-and-spoke arrangement applied to Minnesota. The hub would direct AI algorithms to be performed on selected, relevant images housed in their “natural” sites. Number of screening sites could be readily expanded if desired.

**Figure 2** illustrates a possible state-wide cloud-based network of screening sites. Each site would have a pre-approved informatics construct to allow access to cloud delivered analysis software for already performed LCS images and associated, de-identified clinical data. In this hypothetical example, a possible hub-and-spoke arrangement is applied to Minnesota. The hub would direct AI algorithms to be performed on selected, relevant images housed in their “natural” sites. The number of screening sites could be scaled as required related to the purpose of the analysis.

The proposed digital resource with curated, high-quality imaging/clinical data cases tracked over many years could be readily interrogated efficiently and economically. Patient-specific imaging or clinical data would reside in the screening center and only analysis results would be transmitted back to the cloud-hub. This model of a public service utility comprised of imaging and clinical information could be an exemplar of a 21^st^ century comprehensive medical registry, which maintains transparency in the development and validation of AI tools and objectively evaluates the quality and reliability screening technology in clinical use (13, 14, 36).

### The evolution of imaging data use for public health

While chest CT images include a remarkable amount of already-known clinically relevant information, there could be more actionable information in those images. Access to the already collected images and the associated clinical follow-up information represents an opportunity to economically mine information for the benefit of public health.

Consider that the first indication of ubiquity of atherosclerotic disease emerged from systematic autopsy studies of US military fatalities from the Korean and Vietnam Wars (37, 38). Systematic computational analysis of archived images and clinical data screening chest CT cases could quickly and economically yield novel atherosclerotic information but in large and diverse cohorts of live, LCS participants.

Further, when the first news of high mortality with nesting birds related in part due to pesticide exposure emerged from England in the 1950s, there was no awareness of a comparable problem in North America. Only when a bird survey was done in North America, was a stunning depletion of these birds discovered which was thought in part related to thinning of eggshell (38). LCS imaging can readily detect changes in calcium density in many living tissues including bird shells as well as in bones. LCS images can be evaluated to provide early warning of compromised bone mineral density in already acquired chest CT images (39, 40). Potentially, systematic AI-radiomic analysis of already acquired chest CT screening images could be adapted to serve as an economical and time efficient, 21^st^ century, opportunistic warning or reconnaissance system for evolving biomedical threats.

## Limitations

While the use of large imaging/ data resources has many important potential applications, there are important consideration that limit this approach. Participation in screening incurs consideration with healthy volunteer effect as well as potential for disease misclassification given the inclusion of some degree of heterogenous data quality as well as regional variations in disease pathogenesis. Further, issues with data integrity and privacy are challenges that must be continually monitored and managed with evolving best practices to maintain public confidence with the utility of providing access to screening information and images. Finally, measures to ensure equitable inclusion of images and data from all segments of society are essential to ensure that validated tools can perform reliably as the public expects with a true public health resource.

## Conclusion

LCS images include a remarkable amount of currently useful, clinically relevant information but there is likely even more information of clinical and operational value that is not being used. The imminent availability of millions of LCS images and associated clinical information creates logistical challenges, but the information has huge value. We offer a novel, public utility approach that will accelerate both operational and clinical breakthroughs efficiently and at sustainable, low cost.

## Data Availability

The original contributions presented in the study are included in the article/supplementary material. Further inquiries can be directed to the corresponding author.

## References

[1] Bray F Laversanne M Sung H Ferlay J Siegal RL Soerjomataram I . Global cancer statistics 2022: GLOBOCAN estimates of incidence and mortality worldwide for 36 cancers in 185 countries. CA Cancer J Clin. (2024) 74:229–63. doi: 10.3322/caac.21834, PMID: 38572751

[2] National Lung Screening Trial Research Team . Reduced lung-cancer mortality with low-dose computed tomographic screening. N Engl J Med. (2011) 365:395–409. doi: 10.1056/NEJMoa1102873, PMID: 21714641 PMC4356534

[3] de Koning HJ van der Aalst CM de Jong PA Scholten ET Nackaerts K Heuvelmans MA . Reduced lung-cancer mortality with volume CT screening in a randomized trial. N Engl J Med. (2020) 382:503–13. doi: 10.1056/NEJMoa1911793, PMID: 31995683

[4] Henschke CI Yip R Shaham D Markowitz S Cervera Deval J Zulueta JJ . A 20-year follow-up of the international early lung cancer action program (I-ELCAP). Radiology. (2023) 309:e231988. doi: 10.1148/radiol.231988, PMID: 37934099 PMC10698500

[5] Bonney A Malouf R Marchal C Manners D Fong KM Marshall HM . Impact of low-dose computed tomography (LDCT) screening on lung cancer-related mortality. Cochrane Database Syst Rev. (2022) 2022. doi: 10.1002/14651858.CD013829.pub2/pdf/full PMC9347663 35921047

[6] Tao W Tang X Jiao X Sayani A Zhao J Li W . Effectiveness of interventions for increasing lung cancer screening uptake: A systematic review and meta-analysis of randomized clinical trials. Prev Med. (2026) 203:108489. doi: 10.1016/j.ypmed.2025.108489, PMID: 41448280

[7] Mulshine JL Pyenson B Healton C Aldige C Avila RS Blum T . Paradigm shift in early detection: Lung cancer screening to comprehensive CT screening. Eur J Cancer. (2025) 218:115264. doi: 10.1016/j.ejca.2025.115264, PMID: 39904127

[8] Yip R Mulshine JL Oudkerk M Field J Silva M Yankelevitz DF . Current evidence of low-dose CT screening benefit. Eur J Cancer. (2025) 225:115570. doi: 10.1016/j.ejca.2025.115570, PMID: 40517528

[9] Jiang B Lancaster HL Davies MPA Gratama JC Silva M Han D . AI performance for nodule volume doubling time in the follow-up of the UKLS lung cancer screening study compared to expert consensus and histological validation. Eur J Cancer. (2026) 232:116137. doi: 10.1016/j.ejca.2025.116137, PMID: 41319449

[10] Yip R Jirapatnakul A Avila R Gutierrez JG Naghavi M Yankelevitz DF . Artificial intelligence in low-dose computed tomography screening of the chest: past, present, and future. J Thorac Img. (2026) 41:e0854. doi: 10.1097/RTI.0000000000000854, PMID: 41025216

[11] Califf RM . Now is the time to fix the evidence generation system. Clin Tris. (2023) 20:3–12. doi: 10.1177/17407745221147689, PMID: 36647919

[12] McCollough CH Yu L . CT radiation dose reduction with preserved diagnostic performance: how far have we come over 25 years? AJR Am J Roentgenol. (2026). doi: 10.2214/AJR.25.34450, PMID: 41636571 PMC12969835

[13] Mulshine JL Avila RS Conley E Devaraj A Ambrose LF Flanagan T . The international association for the study of lung cancer early lung imaging confederation. JCO Clin Cancer Info. (2020) 4:89–99. doi: 10.1200/CCI.19.00099, PMID: 32027538 PMC7053806

[14] Lam S Wynes MW Connolly C Ashizawa K Atkar-Khattra S Belani CP . The international association for the study of lung cancer early lung imaging confederation open-source deep learning and quantitative measurement initiative. J Thorac Oncol. (2024) 19:94–105. doi: 10.1016/j.jtho.2023.08.016, PMID: 37595684

[15] Jirapatnakul A Yip R Myers KJ Cai S Henschke CI Yankelevitz D . Assessing the impact of nodule features and software algorithm on pulmonary nodule measurement uncertainty for nodules sized 20 mm or less. Quant Imaging Med Surg. (2024) 14:5057–71. doi: 10.21037/qims-23-1501, PMID: 39022249 PMC11250315

[16] Liang M Tang W Xu DM Jirapatnakul AC Reeves AP Henschke CI . Low-dose CT screening for lung cancer: computer-aided detection of missed lung cancers. Radiology. (2016) 281:279–88. doi: 10.1148/radiol.2016150063, PMID: 27019363

[17] Clark KW Gierada DS Marquez G Moore SM Maffitt DR Moulton JD . Collecting 48,000 CT exams for the lung screening study of the National Lung Screening Trial. J Digit Img. (2009) 22:667–80. doi: 10.1007/s10278-008-9145-9, PMID: 18777192 PMC3043737

[18] Stemmer A Shadmi R Bregman-Amitai O Chettrit D Blagev D Orlovsky M . Using machine learning algorithms to review computed tomography scans and assess risk for cardiovascular disease: Retrospective analysis from the National Lung Screening Trial (NLST). PloS One. (2020) 15:e0236021. doi: 10.1371/journal.pone.0236021, PMID: 32745082 PMC7398499

[19] Pinsky PF Lynch DA Gierada DS . Incidental findings on low-dose CT scan lung cancer screenings and deaths from respiratory diseases. Chest. (2022) 161:1092–100. doi: 10.1016/j.chest.2021.11.015, PMID: 34838524 PMC9005861

[20] Daneshvar N Pandita D Erickson S Snyder Sulmasy L DeCamp M . ACP medical informatics committee and the ethics, professionalism and human rights committee. Artificial intelligence in the provision of health care: an American college of physicians policy position paper. Ann Intern Med. (2024) 177:964–7. doi: 10.7326/M24-0146, PMID: 38830215

[21] Zerilli J Bhatt U Weller A . How transparency modulates trust in artificial intelligence. Patt N Y. (2022) 3:100455. doi: 10.1016/j.patter.2022.100455, PMID: 35465233 PMC9023880

[22] McClelland RL Jorgensen NW Budoff M Blaha MJ Post WS Kronmal RA . 10-year coronary heart disease risk prediction using coronary artery calcium and traditional risk factors: derivation in the MESA (Multi-ethnic study of atherosclerosis) with validation in the HNR (Heinz nixdorf recall) study and the DHS (Dallas heart study). J Am Coll Cardiol. (2015) 66:1643–53. doi: 10.1016/j.jacc.2015.08.035, PMID: 26449133 PMC4603537

[23] Naghavi M Reeves AP Atlas K Zhang C Atlas T Henschke CI . Artificial intelligence applied to coronary artery calcium scans (AI-CAC) significantly improves cardiovascular events prediction. NPJ Digit Med. (2024) 7:309. doi: 10.1038/s41746-024-01308-0, PMID: 39501071 PMC11538462

[24] Yip R Jirapatnakul A Hu M Chen X Han D Ma T . Added benefits of early detection of other diseases on low-dose CT screening. Transl Lung Cancer Res. (2021) 10:1141–53. doi: 10.21037/tlcr-20-746, PMID: 33718052 PMC7947380

[25] Wang BR Edwards R Freiheit EA Ma Y Burg C de Andrade J . The pulmonary fibrosis foundation patient registry. Rationale, design, and methods. Ann Am Thorac Soc. (2020) 17:1620–8. doi: 10.1513/AnnalsATS.202001-035SD, PMID: 32776789 PMC12057645

[26] Bajcsy P Bhattiprolu S Börner K Cimini BA Collinson L Ellenberg J . Enabling global image data sharing in the life sciences. Nat Methods. (2025) 22:672–6. doi: 10.1038/s41592-024-02585-z, PMID: 40155720 PMC12617600

[27] Available online at: https://www.cms.gov/priorities/innovation/innovation-models/participant/health-care-innovation-awards/bronx-rhio (Accessed January 16, 2026).

[28] Obuchowski NA Buckler A Kinahan P Chen-Mayer H Petrick N Barboriak DP . Statistical issues in testing conformance with the quantitative imaging biomarker alliance (QIBA) profile claims. Acad Radiol. (2016) 23:496–506. doi: 10.1016/j.acra.2015.12.020, PMID: 26898527 PMC4831211

[29] Rydzak CE Armato SG Avila RS Mulshine JL Yankelevitz DF Gierada DS . Quality assurance, and quantitative imaging biomarkers in low dose CT lung cancer Screening. Br J Radiol. (2018) 91:20170401. doi: 10.1259/bjr.20170401, PMID: 28830225 PMC6350468

[30] Faden RR Beauchamp TL Kass NE . Learning health care systems and justice. Hast Cent Rep. (2011) 41:3. doi: 10.1002/j.1552-146x.2011.tb00105.x, PMID: 21845906

[31] Larson DB Magnus DC Lungren MP Shah NH Langlotz CP . Ethics of using and sharing clinical imaging data for artificial intelligence: A proposed framework. Radiology. (2020) 295:675–82. doi: 10.1148/radiol.2020192536, PMID: 32208097

[32] Ricks M Sitaraman G Welton S Menand L . “Networks, platforms, and utilities: law and policy”. In: Faculty books, vol. 349. New York City: Columbia Law School. (2022). Available online at: https://scholarship.law.columbia.edu/books/349 (Accessed February 28, 2026).

[33] Available online at: https://en.wikipedia.org/wiki/Maternal_mortality_in_the_United_States (Accessed January 16, 2026).

[34] Available online at: https://www.health.ny.gov/regulations/hcra/ (Accessed January 16, 2026).

[35] Nelson DB Moniz MH Davis MM . Population-level factors associated with maternal mortality in the United States, 1997-2012. BMC Public Health. (2018) 18:1007. doi: 10.1186/s12889-018-5935-2, PMID: 30103716 PMC6090644

[36] Avila RS Krishnan K Obuchowski N Jirapatnakul A Subramaniam R Yankelevitz D . Calibration phantom-based prediction of CT lung nodule volume measurement performance. Quant Imaging Med Surg. (2023) 13:6193–204. doi: 10.21037/qims-22-320, PMID: 37711774 PMC10498266

[37] Joseph A Ackerman D Talley JD Johnstone J Kupersmith J . Manifestations of coronary atherosclerosis in young trauma victims--an autopsy study. J Am Coll Cardiol. (1993) 22:459–67. doi: 10.1016/0735-1097(93)90050-b, PMID: 8335815

[38] Webber BJ Seguin PG Burnett DG Clark LL Otto JL . Prevalence of and risk factors for autopsy-determined atherosclerosis among US service members, 2001-2011. JAMA. (2012) 308:2577–83. doi: 10.1001/jama.2012.70830, PMID: 23268516

[39] Peakall DB . DDE-induced eggshell thinning: an environmental detective story. Environ Rev. (1993) 1:13–20. doi: 10.1139/a93-002, PMID: 36563491

[40] Jiang W Huang J Wu N Liu J Liang J Li S . Thoracic vertebral bone mineral density measured by quantitative computed tomography is associated with fracture risk in lung cancer screening populations: a prospective cohort study. Front Endocrinol Lausanne. (2025) 16:1672551. doi: 10.3389/fendo.2025.1672551, PMID: 41323983 PMC12657172

